# Cognitive Impairment and Dementia in Atrial Fibrillation: A Population Study of 4.3 Million Individuals

**DOI:** 10.1016/j.jacadv.2023.100655

**Published:** 2023-10-25

**Authors:** Sheng-Chia Chung, Martin Rossor, Ana Torralbo, Cai Ytsma, Natalie K. Fitzpatrick, Spiros Denaxas, Rui Providencia

**What is the clinical question being addressed?**

We investigated the risk of MCI after AF diagnosis in the United Kingdom.

**What is the main finding?**

Our study showed that AF was associated with a 45% increase on the risk of MCI and that cardiovascular risk factors and multicomorbidity appear to associate with this outcome.

Mild cognitive impairment (MCI) is an early stage in cognitive function decline that is greater than that observed in healthy aging but of insufficient severity to fulfil the criteria of dementia. Causes for MCI are heterogeneous, and while MCI may be reversible in some individuals, it may reflect an early dementia-associated disease processes, with annual conversion rate of ≈20%.^1^ Factors associated with the development, or protection from, MCI in atrial fibrillation (AF) patients, and subsequent development of dementia, have not been fully elucidated.

We investigated the association of AF with MCI and subsequent dementia using routinely collected UK primary electronic health record (EHR) data.

We used the UK-based linked EHR of 4.3 million individuals between January 1, 1998, and May 31, 2016. All individuals with incident AF were included, and the index date was defined as the date of the first recorded AF diagnosis. For each case, we randomly selected 1 AF-free individual as a control from the study cohort who was matched to the sex and age at diagnosis of the individual with incident AF. AF was defined as I48 of the International Statistical Classification of Diseases-10th Revision (ICD-10) and corresponding Read version 2 terms (Read V2) from the Clinical Practice Research Datalink.^2^ The primary outcome of the study was the incidence of MCI, defined as ICD-10 codes G31.8 and F06.7 and corresponding Read terms. Follow-up ceased with death, end of registration with the practice, cessation of the contribution of data to the CPRD, or end of the study period. We studied the association between AF and MCI in relevant subgroups including age at AF diagnosis, sex, socioeconomic categories, stroke and treatment of digoxin, oral anticoagulants, and amiodarone treatment. We investigated the association between AF and MCI in Cox proportional hazards model controlled for competing risk. Competing events (eg, death) were treated as censored observations. Adjustment was performed for age, sex, calendar year at study entry, socioeconomic status, smoking, hypertension, diabetes, obesity, hypercholesterolemia, hearing loss, thyroid disease, depression, atherosclerotic heart disease, peripheral artery disease, heart failure, stroke, cancer, chronic kidney disease, liver disease, and chronic obstructive pulmonary disease. We applied the same method to study the subsequent dementia incidence in participants developed MCI.

**Figure 1:**
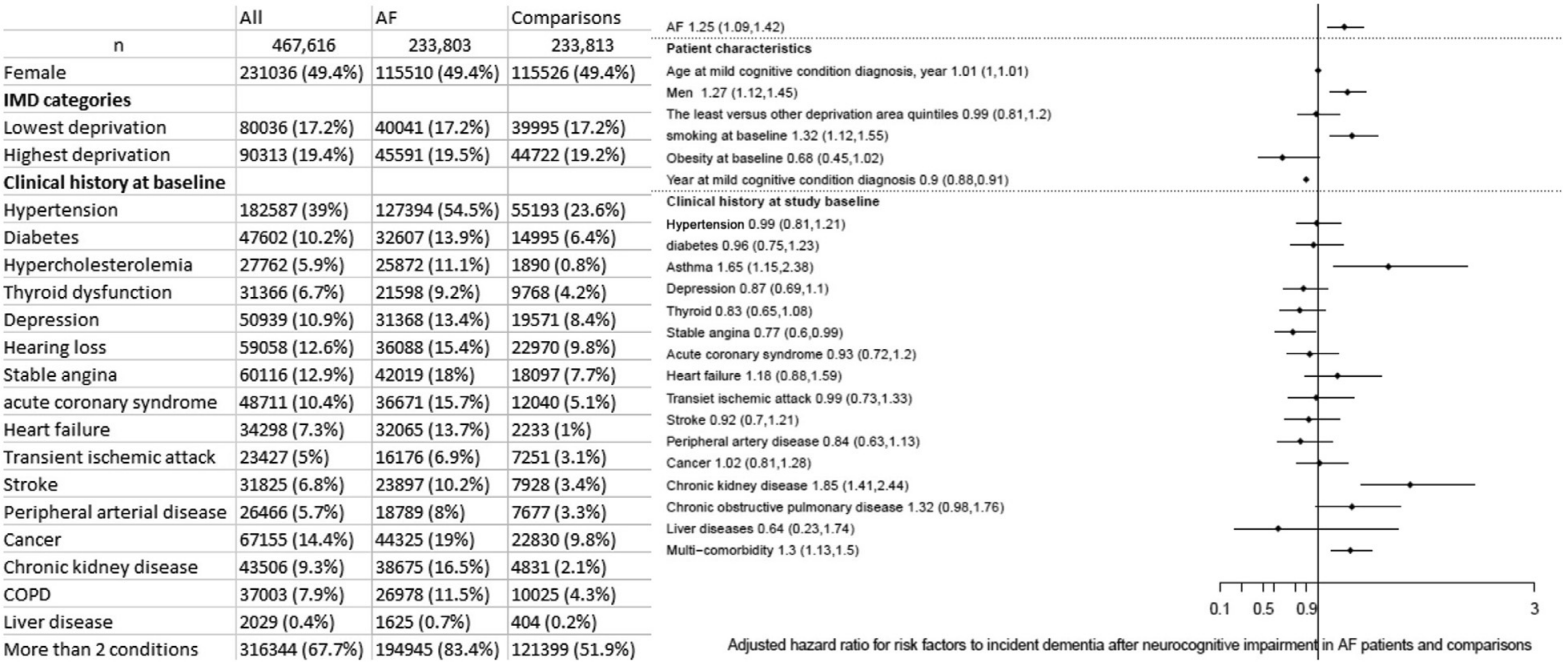
Study Population and Risk Factors for Incident Dementia Following a Diagnosis of Mild Cognitive Impairment (Left) Characteristics of participants with AF and age- and sex-matched controls; (right) adjusted HR associated with each risk factor for the incidence of dementia after the diagnosis of mild cognitive impairment. AF = atrial fibrillation; COPD = chronic obstructive pulmonary disease; IMD = Index of Multiple Deprivation.

We analyzed data for 4,309,245 eligible individuals in the United Kingdom and identified 233,833 (5.4%) individuals with incident AF and a total of 233,747 non-AF (Figure 1). The mean age was 74.2 years in both AF and non-AF patients. During a median of 5.3 years of follow-up, there were 4,269 total incident MCI cases from both AF and non-AF patients. Persons with AF had a higher risk of MCI than non-AF individuals, with an adjusted HR of 1.45 (95% CI: 1.35-1.56). Besides AF, risk factors such as older age, female sex, higher socioeconomic deprivation, clinical history of depression, stroke, and multimorbidity were associated with a greater risk of MCI (risk ratio ranging from 1.08 (age in years) to 1.44 (history of depression at baseline), all *P* < 0.001).

Analyses showed similar results in the population stratified by age at AF diagnosis, sex, socioeconomic deprivation, and history of stroke. Patients with incident AF receiving digoxin treatment did not experience an increased risk of MCI (HR: 0.97; 95% CI: 0.53-1.78). Similarly, whereas MCI risk was higher in AF patients not receiving oral anticoagulant treatment and amiodarone treatment, AF patients receiving oral anticoagulant treatment and amiodarone treatment were not at risk of MCI.

Among individuals who developed MCI, there were 1,117 dementia diagnosis on or after MCI diagnosis during the study period. Individuals with AF also associated with higher risk of dementia among those who developed MCI (multiple-adjusted HR: 1.25 (95% CI: 1.09-1.42). Risk factors associated with subsequent dementia risk were sex, smoking, asthma, chronic kidney disease, and multicomorbidity (Figure 1).

Our study showed that AF was associated with a 45% increased risk of MCI in a real-world nationally representative cohort. The results showed that age, greater sociodemographic deprivation, and clinical history of stroke were associated with a higher risk of MCI but did not modify the association between AF and MCI. Both AF and MCI were frequently diagnosed in individuals aged over 74 years when multicomorbidity was present, and we found diabetes, hypercholesterolemia, depression, and peripheral artery disease are also associated with an elevated risk of MCI. Progression from MCI to dementia appears to be, at least partially, mediated by cardiovascular risk factors and the presence of multiple comorbidities. Silent brain infarcts are frequent in the AF population and have been previously associated with cognitive dysfunction.^3^^,^^4^

Some limitations need to be acknowledged: as for all electronic health record studies, possible lack of data granularity and level of detail in the dataset is a potential limitation. Risk of unmeasured risk factors or comorbidities is also something that needs to be borne in mind. We hope to have minimized this issue with detailed characterization of the sample and presentation of 16 frequently associated comorbidities. Underreporting of cognitive impairment, due to the subtility/unspecificity of symptoms and sub-optimal use of cognitive testing is a problem for research for the field, also identified for EHR.^5^ However, ICD-10 have been previously used in MCI studies with acceptable performance.^6^ Finally, an increase in risk of MCI was not observed in individuals with AF receiving digoxin, or amiodarone treatment, with risk in these patients being comparable to their non-AF peers. However, the observational design of this study and very wide confidence interval for these subgroups of patients (accounting for only 10%-20% of the AF sample) does not allow us to make any solid inferences about causality, a potential protective role of these drugs or unmeasured confounders.

Our findings emphasize the association of multicomorbidity and cardiovascular risk factors with AF development of MCI and progression to dementia in the AF population. These data provide support to the previous hypothesis of integrated AF care^7^ (combining anticoagulation, symptom, and comorbidity-management) as a way of preventing cognitive deterioration and progression to dementia, highlighting the need for a confirmatory clinical trial.
